# Amyloid-beta peptide toxicity in the aged brain is a one-way journey into Alzheimer’s disease

**DOI:** 10.3389/fnagi.2025.1569181

**Published:** 2025-04-30

**Authors:** Georgia Culley, Alexandre Henriques, Delphine Hardy, Alexandre Wojcinski, Adrien Chabert, Bilal El Waly, Philippe Poindron, Noelle Callizot

**Affiliations:** Neuro-Sys Vivo, Gardanne, France

**Keywords:** amyloid toxicity, aging, Alzheimer’s, spreading, neurodegeneration, tau, neuroinflammation, oligomers

## Abstract

Aging is the primary risk factor for Alzheimer’s disease (AD), and the aging brain shares many characteristics with the early stages of AD. This study investigates the interplay between aging and amyloid-beta (Aβ) induced pathology. We developed an AD-like *in vivo* model, using the stereotactic injection of Aβ_1–42_ oligomers into the hippocampi of aged mice. Cognitive impairments were assessed using a Y maze. Immunohistochemical and protein analyses were conducted to evaluate neuronal survival, synaptic function and number, levels of tau hyperphosphorylation, microglial activation, autophagy, and mitochondrial function. We compared baseline aging effects in young adult (3 months) and aged (16-18 months) healthy mice. We found that aged mice displayed significant deficits in working memory, synaptic density and neurogenesis, and an increased basal inflammation. In response to acute injury to the hippocampus with Aβ oligomer injection, aged mice suffered sustained deficits, including impaired cognitive function, further reduced neurogenesis and synaptic density, increased microglial activation, astrogliosis, mitochondrial stress, and lysosomal burden. Furthermore, in the weeks following injury, the aged mice show increased amyloid accumulation, microglial activation and phosphorylated tau propagation, expanding from the injection site to adjacent hippocampal regions. In contrast, the young adult mice exhibited only acute effects without long-term progression of pathology or neurodegeneration. We conclude that the aging brain environment increases susceptibility to an acute Aβ injury, creating fertile soil for the progression of AD, whereas younger brains are able to overcome this injury. The processes of aging should be considered as an integral factor in the development of the disease. Targeting aging mechanisms may provide new strategies for AD prevention and treatment, as well as for other neurodegenerative diseases.

## Introduction

As Alzheimer’s disease (AD) mainly affects people over the age of 65, and indeed age is the primary risk factor (Kawas et al., 2000), it is relevant to focus on how the general characteristics of brain aging contribute to the disease’s initiation and progression. Could addressing the root causes of aging itself, therefore, be a key strategy in preventing or slowing the onset of AD and other neurodegenerative diseases?

The main established pathological features of AD are extracellular amyloid-beta (Aβ) plaque deposits, intracellular neurofibrillary tangles, and progressive synaptic loss followed by neuronal death. Subsequently, many cellular key players of the pathology have been identified, such as impairments in synaptogenesis, mitochondrial functions, autophagy, as well as increased activation of microglia, all of which correlate with the general dysfunction that develops in the aging neuron. As these defects tend to be detected earlier in the pathology of AD than the appearance of Aβ plaques and neurofibrillary tangles, they present interesting targets for disease prevention, in comparison to later inventions that could only slow the development of symptoms.

Although Aβ is present and physiologically important (including both Aβ_1–40_ and Aβ_1–42_), for example for synaptic plasticity, in healthy brains of all ages (Cai et al., 2023), the aging brain tends to have a higher proportion of Aβ_1–42_, which is more prone to forming oligomers and fibrils. Aβ_1–42_ oligomers, the most toxic entity, disrupt synaptic plasticity, activate Glycogen synthase kinase-3 β (GSK3β kinase, involved in tau phosphorylation) and drive neuronal death (Lambert et al., 1998, Vossel et al., 2015). In AD patients, the shift from Aβ_1–40_ to Aβ_1–42_ is evident (Hampel et al., 2021).

Aging leads to a reduction in the number and size of synapses (Peters et al., 2008). Synaptic loss in regions such as the hippocampus and cortex correlates strongly with memory impairment and other cognitive deficits. In AD, synaptic atrophy is prominent, leading to impaired neuronal communication and cognitive decline (Shankar and Walsh, 2009).

Neurons are particularly susceptible to the effects of aging because of their much greater energy demands. Studies on primates and rodents have shown a reduction in the activity of all mitochondria in aged brains, leading to a bioenergetic deficit (Bowling et al., 1993; Long et al., 2009). With less energy available, neuronal function is compromised; a major factor in the physiological cognitive decline typical of aging. Oxidative stress–driven metabolic disruption affects proteins and nucleic acids and can contribute to the pathogenesis of AD (Dhapola et al., 2024). Furthermore, dysfunctional mitophagy and mitogenesis are related to various neurodegenerative diseases (Mangrulkar et al., 2023).

The fact that neurons can live for more than a century without regeneration makes their cellular repair, recycling, and clearance mechanisms particularly important. Autophagy-lysosomal pathways and mitophagy, essential for normal brain function, are known to decline with aging (Nixon et al., 2008; Carmona-Gutierrez et al., 2016). Recent research has highlighted the critical role of impaired autophagy in the progression of AD. Studies have shown that deficits in autophagy occur early in AD, contributing to the accumulation of senile plaques and neurofibrillary tangles (Orr and Oddo, 2013).

In normal aging, microglia, the brain’s primary immune cells, undergo several changes that affect their function. These cells exhibit a gradual shift from a homeostatic state to a more pro-inflammatory state, even in the absence of disease (Javanmehr et al., 2022). This shift includes a reduction in their ability to perform essential tasks, such as immune surveillance, debris clearance, and response to injury. Interestingly, aging microglia are characterized by alterations in morphology, phagocytosis, metabolism, and inflammatory phenotype, which appear to play both protective and detrimental roles in maintaining brain homeostasis and preserving their ability to respond to insults (Antignano et al., 2023; Passarella et al., 2024). Additionally, there is an increase in the production of pro-inflammatory cytokines, contributing to a low-grade, chronic inflammatory state known as “inflammaging.” In AD, the microglia become chronically activated, leading to a sustained release of pro-inflammatory cytokines that exacerbate neuroinflammation and neuronal damage. Additionally, microglia in AD show impaired phagocytic function, so are less effective at clearing debris. This accumulation further drives disease progression (Nizami et al., 2019).

In addition to amyloid-targeting therapies, there is a growing focus on addressing earlier cellular dysfunctions, such as impaired autophagy, which are evident in both aging and AD brains. Recent research has demonstrated that enhancing autophagy can reduce toxic protein aggregates and improve cognitive function in AD models.

In this study, we investigated whether, given the right “soil,” a suitable environment, an initial insult (Aβ_1–42_ oligomers injected into the hippocampi) could trigger a neurodegenerative process. We compared different ages choosing “young adult animals” (3 months) and elderly animals (16-18 months). We show that in aged animals, this stress induces damage, not only around the stress site but also in other areas. This ultimately leads to a self-perpetuating degenerative process that would never settle down. In young animals, preserved homeostasis ultimately helps contain the stress, demonstrating resilience and intact cellular mechanisms capable of overcoming the initial Aβ injury. We conclude that the accumulated impairments in aged brains result in the increased toxicity and the progression of neuronal damage.

## Materials and methods

### Animal housing

Male C57BL/6 mice (aged 3 or 16–18 months) were obtained from Charles River Laboratories. The animals were housed in the Neuro-Sys VIVO animal facility under a reversed light/dark cycle and acclimated for at least 5 days prior to surgical procedures. Enrichment materials (e.g., nesting paper and cardboard rolls) were provided in the cages, which housed 2–4 animals each to promote social interaction. Food and water were available *ad libitum*. To minimize stress during subsequent behavioral testing, mice were handled regularly, and their body weight was monitored daily. All experiments were carried out in accordance with the National Institutes of Health Guide for the Care and Use of Laboratory Animals and followed current European Union regulations (Directive 2010/63/EU). The study was approved by the local Animal Ethics Committee of Marseille (CEEA14). Agreement number: B1301337.

### Aβ_1–42_ peptide preparation

The Aβ_1–42_ preparation followed the protocol established by Callizot et al. (2013, 2021). Briefly, Aβ_1–42_ peptide was dissolved in the vehicle to achieve an initial concentration of 100 μM. The solution was gently agitated for 3 days at 37°C in darkness. (An example WES trace to characterize the preparation can be found in Supplementary Figure 1).

### Surgical procedures

Mice were anesthetized with isoflurane (4% for induction) using an induction chamber connected to a vaporizer and oxygen concentrator. During the surgery, anesthesia was maintained with isoflurane (2%) delivered via a face mask. Lidocaine (1 mg/kg, subcutaneous) was administered cranially for local anesthesia before craniotomy. Additionally, buprenorphine (0.1 mg/kg, subcutaneous) was administered 30 min prior to surgery for analgesia. Rectal temperature was continuously monitored and maintained at 37°C with a heating mat, and ocular gel (Lubrithal) was applied to prevent corneal desiccation. Anesthetic depth was verified through palpebral reflex, vibrissae movement, and absence of response to tail and toe pinches before initiating surgery.

The skull was exposed, and craniotomy was performed at the designated coordinates. Aβ_1–42_ preparation was injected bilaterally (see Supplementary Figure 2 for illustration) into the CA1 hippocampal region (anterior-posterior: –2.0 mm; medio-lateral: ± 1.8 mm) at three depths [dorso-ventral (DV)]:

Stratum oriens: DV: –1.3 mm.

Stratum pyramidale: DV: –1.5 mm.

Stratum radiatum: DV: –1.7 mm.

A total of 3 μL of Aβ_1–42_ solution (1 μL per site, 100 μM, ∼15 μM oligomers (AβO), measured by automated protein quantification using the Simple Western (WES) system) or vehicle was injected into each hemisphere using a Hamilton syringe connected to an Elite Nanomite syringe pump (0.2 μL/min). Between each injection, the needle was left in place for 2 min and then withdrawn slowly to minimize tissue damage. Sham animals underwent identical surgical procedures but received intracranial injections of vehicle (0.9% NaCl) at the same coordinates.

Following surgery, mice recovered under a heating lamp before being returned to their cages.

Daily observations and scoring were recorded on follow-up sheets to monitor postoperative health for 5 days after surgery.

### Y maze behavioral assessment (forced alternation)

One week prior to surgery, mice underwent a habituation session in the Y maze. During this session, each mouse was allowed to freely explore all three arms of the maze for 5 min to acclimate to the testing environment. The Y maze dimensions were as follows: external arm length, 36 cm; internal arm length, 34.5 cm; external arm width, 7.6 cm; internal arm width, 6 cm; external height, 15.5 cm; internal height, 15 cm. Each arm of the maze contained distinct visual cues. All tests were conducted under standard lighting conditions. Mice were acclimated to the testing room for 1 h prior to each test session.

Short-term spatial memory was assessed using the forced-alternation Y maze test. It was conducted on post-surgery days 14, 21, or 28. Testing consisted of two phases:

- Two-Arm Exploration: Mice were allowed to explore two arms of the Y maze for 5 min, with the third arm closed. Following this phase, mice were placed in an empty cage to rest for 3 min. Between trials, the maze was cleaned with acetic acid to neutralize any odors.

- Three-Arm Exploration: Mice were subsequently allowed to freely explore all three arms of the maze for 5 min.

All trials were recorded using a video camera and analyzed with the EthoVision system (Noldus). Locomotor activity and the time spent in each arm were automatically quantified for each mouse.

#### BrdU administration

Bromodeoxyuridine (BrdU) was solubilized in the vehicle at a final concentration of 100 mg/kg in saline (0.9% NaCl) and kept at 4°C, protected from light. For mice assigned to neurogenesis analysis by immunohistochemistry (IHC), BrdU administration began after the last behavioral analysis. A total of 5 administrations of BrdU (100 mg/kg/d, i.p.), were performed daily in the 5 days preceding sacrifice.

### Protein analysis using WES; PSD95 and Aβ

Levels of Postsynaptic Density Protein 95 (PSD95) and Aβ (measured by the 6E10 antibody) were assessed in hippocampal tissue by automated protein analysis. Briefly, tissues were micro-dissected and lysed with a defined buffer lysis consisting of CelLyticMT reagent with 1% protease and phosphatase inhibitor cocktail (60 μL per well). Lysates were stored at –80°C and processed at + 4°C. For each condition, the quantity of proteins was determined using the micro kit BCA (Pierce). Protein analysis was performed using WES™ automated Western blotting and analysis (ProteinSimple^®^). All reagents (ref: SM-W002, except primary antibodies) and secondary antibodies (ref: DM-001 or DM-002) were provided by ProteinSimple^®^. They were prepared and used according to manufacturer’s recommendations for use on WES™ (ProteinSimple, San Jose, CA).^1^

Capillaries, samples, antibodies, and matrices were then loaded inside the instrument. The quantity of protein loaded was set to 0.5-2 mg/mL. The simple Western was run with capillaries filled with separation matrix, stacking matrix and protein samples. Next, capillaries were incubated 2 h with primary antibodies (see Supplementary Table 1), at room temperature (23 ± 3°C).

Capillaries were washed and then incubated with horseradish peroxidase (HRP)-conjugated secondary antibodies for 1 h, at room temperature. After removal of unbound secondary antibody, the capillaries were incubated, at room temperature, with the luminol-S/peroxide substrate and chemiluminescent signal was collected using the Charge-Coupled Device (CCD) camera of WES™ with six different exposure times (30, 60, 120, 240, 480, and 960 s). Data analysis was performed using the Compass Software (ProteinSimple) on WES™. For 6E10, peaks were automatically measured between 40 and 230 kDa (see Supplementary Figure 4 for example of computer generated trace).

### Immunohistochemistry; NeuN, Iba1, BrdU, DCX, pTau, GFAP, Lamp2, PINK1, CytoC

Serial coronal 20 μm-thick sections of the hippocampal area were cut using a cryostat and stored at –20°C. Free-floating sections were incubated in tris-buffered saline (TBS) with 0.25% bovine serum albumin (BSA), 0.3% Triton X-100 and 1% goat serum, for 1 h at room temperature. Brain sections were then incubated overnight at room temperature with primary antibodies for the following targets (see Supplementary Table 1); Neuronal nuclei marker (NeuN, marker of mature neurons), ionized calcium-binding adaptor molecule 1 (Iba1, marker of microglia), BrdU (marker of proliferating cells), doublecortin (DCX, marker of immature neurons), AT100 (antibody to hyperphosphorylated tau or pTau), glial fibrillary acidic protein (GFAP, marker of astrocytes), lysosome-associated membrane protein 2 (Lamp2, marker of lysosomes), PTEN-induced kinase 1 (PINK1, marker of mitophagy) and cytochrome c (CytoC, marker of mitochondrial stress). These antibodies were revealed using secondary antibodies coupled with Clear Fluor™ or Alexa Fluor, at the dilution 1/500, in TBS with 0.25% BSA, 0.3% Triton X-100 and 1% donkey serum. Cell nuclei were counterstained with DAPI or Hoechst.

Images were acquired with either a confocal laser-scanning microscope (LSM 900 with Zen software, Zeiss) or Axioscan7 (Zeiss). Tile size = 320 μm^2^ (see Supplementary Figure 2 for schematic illustration). Automated image analysis was performed with MetaXpress^®^ (Molecular Devices) software. Analysis of Axioscan images was performed approximately 150 μm from the injection site.

### Statistical analysis

All values are expressed as mean ± SEM (standard error of the mean). Statistical analysis was performed by unpaired *t*-test, one-way or two-way ANOVA, depending on the format of the experiment (i.e., number of groups), followed by a Fisher’s LSD test, using GraphPad prism. *p* < 0.05 was considered significant. N number, type of statistical test, and raw value to calculate 100% are included in the figure legends for each graph. Power of significance (*p < 0.05, **p < 0.01, ***p < 0.001 and ****p < 0.0001) is provided for each result in the results section.

## Results

### Accumulated deficits with normal aging

**FIGURE 1:**
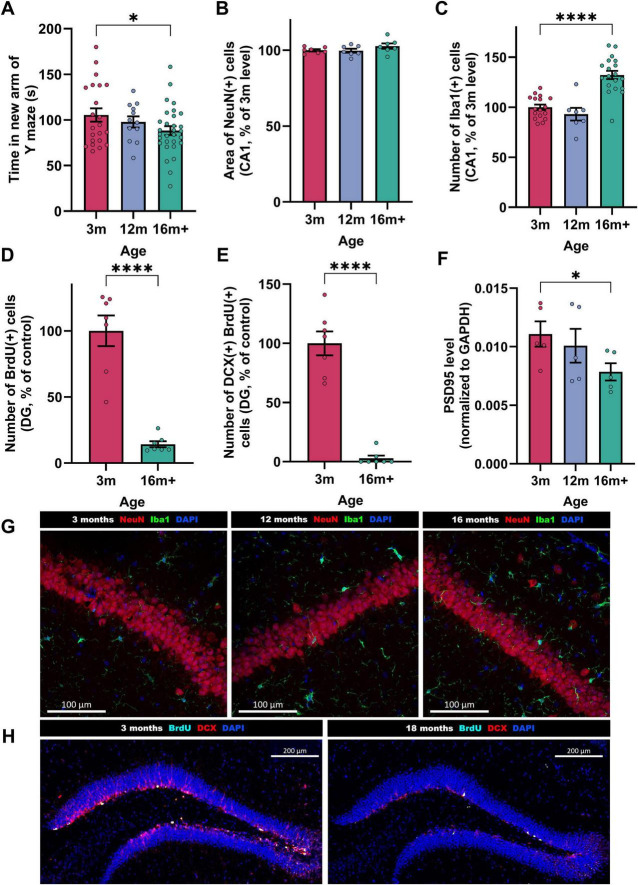
Features of brain-aging in un-injured mice aged 3, 12 or 16 + months. Behavioral readout; **(A)** short term spatial memory performance (Time spent in the new arm of the Y maze, *n* = 12-30). Histological readouts; **(B)** mature neurons [NeuN + area, *n = 7*, as proportion of 3 m mean (100% = 49,630 ± 706 μm^2^/field/animal)] in CA1, **(C)** activated microglia [Iba1 + cell number, *n* = 7−19, normalized to 3 m mean where 100% = 9.27 ± 0.3 cells/field/animal] in CA1, **(D)**, proliferating cells [BrdU + cells, *n = 7*, normalized to 3 m mean where 100% = 14.65 ± 1.7 cells/field/animal] in substantia granulosa of DG. **(E)** proliferating cells differentiating into neurons (BrdU + DCX + , *n = 7*, normalized to 3 m mean where 100% = 1.89 ± 0.2 cells/field/animal) in DG. Biochemical readout; **(F)** Synaptic density of synapses (PSD95/GAPDH ratio, *n* = 5) in the hippocampus. Each bar represents the mean ± SEM, one-way ANOVA followed by Fisher’s test (to compare 3 ages) or unpaired *t*-test (to compare 2 ages). *p < 0.05, **p < 0.01, ***p < 0.001 and ****p < 0.0001. Representative immunostaining images: **(G)** NeuN (red), Iba1 (green), and DAPI (blue) at 3, 12, and 16 + months. Scale bars indicate 100 μm. **(H)** BrdU (cyan), DCX (red) and DAPI (blue) staining in the DG at 3, 12, and 16 + months. Scale bars indicate 200 μm.

Beginning by characterizing healthy (un-injured) mice, 3 and 16-18 month-old mice were selected as our ages of interest. The 3 month-old mice, considered adults having reached sexual maturity, are frequently used in AD models (Forner et al., 2021; Jawhar et al., 2012). Although 12-month-old mice are often classified as “old” in the literature, our data on un-injected (i.e., healthy) mice indicate that significant changes continue to occur beyond this age (Figure 1). We showed that at 16–18 months, mice performed significantly worse in spatial working memory tests (Figure 1A; average time in new arm for young = 105 s, for aged = 88 s, *p* = 0.04). While the amount of mature neurons in the CA1 region of the hippocampus remained stable (Figure 1B), neuroinflammation, or the proliferation of microglia, increased with age (Figure 1C; 16 m + mice have 32% more Iba(+) activated microglia in hippocampal CA1 compared to 3 m, *p* < 0.0001). As microglia become activated with increasing age [producing reactive oxygen species (ROS) and secreting pro-inflammatory cytokines], they also lose the ability to phagocytose (Woodburn et al., 2021).

In addition, a strong reduction of neurogenesis was observed in the subgranular zone of the dentate gyrus (DG), as seen in Figures 1D,E [86% fewer BrdU(+) cells in DG of 16 + vs. 3 m brains, of which 97% fewer are DCX(+), i.e., differentiating into neurons, *p* < 0.0001]. The cognitive deficits observed were associated with a significant reduction of synapses (Figure 1F; 29% lower hippocampal PSD95 levels). Although the aged mice are still healthy enough to tolerate surgery and behavioral testing, they show substantial differences in the parameters explored in this study, making them an ideal model.

### AβO-injected animals; Young adult (3 months) vs. Aged mice (16 + months)

#### Y maze performance in AβO-injected animals (young adult vs. aged mice)

**FIGURE 2:**
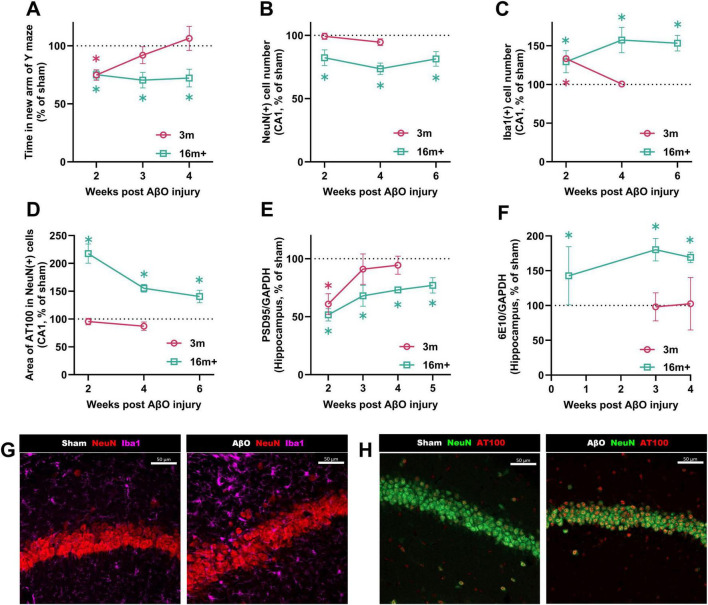
Kinetics post-injection in Aβ-injected mice at different ages compared to sham-injected controls. Comparison over time in 16 + month-old and 3-month-old mice: **(A)** Time spent in the new arm of the Y maze (*n* = 10-21, sham at week 2; 3 m = 131.1 ± 11.3 s, 16 m = 123.2 ± 6.4 s). **(B)** NeuN + cell count (*n* = 4-8, sham at week 2; 3 m = 41.0 ± 1.5 cells). **(C)** Iba1 + cell count (*n* = 5-8, sham at week 2; 3 m = 16.0 ± 2.0, 16 m = 11.8 ± 1.3). **(D)** Area of pTau (AT100) per NeuN + cell (*n* = 5-16, sham at week 2; 3 m = 782.5 ± 30.2 μm^2^, 16 m = 618.2 ± 71.0 μm^2^). **(E)** PSD95 levels relative to the housekeeping gene GAPDH in the hippocampus (*n* = 4-5, sham at week 2; 3 m = 0.019 ± 0.002, 16 m = 0.0109 ± 0.0004). **(F)** 6E10 levels relative to the housekeeping gene GAPDH in the hippocampus (*n* = 5-7, sham at week 4; 3 m = 0.016 ± 0.003, 16 m = 0.0026 ± 0.0002). Data are presented as a proportion of the sham level for each group. Each bar represents the mean ± SEM, two-way ANOVA followed by Fisher’s test. *p < 0.05. Representative immunostaining images of NeuN, Iba1, and pTau (AT100) in sham and Aβ-injected aged mice at 4 weeks post-injection. **(G)** NeuN (red)/Iba1 (pink). **(H)** NeuN (green)/AT100 (red). Confocal microscopy of the CA1 region, closely adjacent to injection site. Left images; sham-injected mice, right images; Aβ-injected mice. Scale bars indicate 50 μm.

The impact of AβO injection on short-term spatial working memory differed markedly between young and aged mice. At 2 weeks post-injection, AβO-injected mice performed significantly worse than sham-injected controls, in both the 3 month and 16 + month groups (Figure 2A; both at 75% of age-control sham). The performance of 3 month mice gradually improved from week 3, and was equal to that of sham-treated controls by week 4, illustrating the capacity for young adult mice to recover short-term spatial working memory functions after the initial AβO-induced impairment. In contrast, 16 month + AβO-injected mice showed sustained impairments on the Y maze task, with performance remaining significantly lower than sham-treated animals up to 4 weeks post-injection (72.2% of sham, *p* = 0.005), highlighting the diminished resilience of the aged brain to regain spatial working memory function once impaired.

#### Differing effects of AβO-injection over time—(young adult vs. aged mice)

NeuN, a marker of mature neurons, was assessed in the CA1 region to assess the impact of AβO on neuronal density. For the younger adult groups, at 2 or 4 weeks post-injection, there was no difference in the number of mature neurons between sham-injected and AβO-injected mice (Figure 2B; 99% of sham at week 2, *p* = 0.9; 95% of sham at week 4, *p* = 0.3). For the aged mice, however, there were significantly fewer mature neurons in the CA1 area of the brain in the AβO injured group, over the 2-6 week time period investigated (82%, *p* = 0.02 at week 2; 74%, *p* < 0.0001 at week 4; 81%, *p* = 0.001 at week 6).

Similarly, whilst young adult mice recover from the toxic effects of AβO on synaptic density by 4 weeks post-injection (Figure 2E; PSD95 levels at 94% of sham), the synaptic density of aged animals remains significantly impaired (PSD95 levels at 73% of sham at week 4, *p* < 0.0001; see Supplementary Figure 3 for example WES trace).

Microglia are well known contribute to neurodegeneration in AD through the release of toxic substances and regulation of synaptic function (Hong et al., 2016). We investigated how the injection of AβO might yield differing results depending on the age of the subject. Iba1, reflecting the number of microglia, was therefore assessed by IHC. Microglial proliferation (Figure 2C; Iba1 +) was increased 2 weeks after amyloid injection in the CA1 of both 3 (133% of sham, *p* < 0.0001) and 16 + (130% of sham, *p* = 0.0008) month mice. However, at 4 weeks post-injection, no inflammation was observed in 3 month brains (compared to sham, 101%, *p* = 0.9). This complete recovery of inflammation and synaptic density in young animals at 4 weeks after the lesion paralleled the complete recovery of cognitive function in the Y maze. In clear contrast, in 16 + month brains, the inflammation remains elevated up to 6 weeks post-injection (Figure 2C; 157% at week 4, *p* = 0.0008; 153% at week 6, *p* < 0.0001), demonstrating a failure to resolve the inflammatory response to the initial injury.

#### Amyloid beta and phosphorylated tau protein levels in AβO-injected animals (3 vs. 16 + month aged mice)

As the accumulation and aggregation of Aβ and hyperphosphorylated tau proteins are hallmark features of AD pathology, we investigated the levels of these proteins by automated western blot (for Aβ, detected by the antibody 6E10) or by immunohistochemistry (for pTau, by the AT100 antibody). Showing a stark age-dependent response, phosphorylated tau protein (AT100) was significantly increased in the CA1 of 16 + month AβO-injected mice, remaining elevated up to 6 weeks after the lesion (Figure 2D; 140% of sham, *p* = 0.01 at week 6); by contrast no AT100 signal was observed in the CA1 of 3 month brains at 2 or 4 weeks after AβO injection (Figure 2D; 96% of sham, *p* = 0.6; 87% of sham, *p* = 0.2 at week 4).

Similarly, the Aβ accumulation varied greatly with age. At 4 weeks after the injection of AβO, Aβ protein levels of young mice, detected in the whole hippocampus, were no different to sham-treated animals (Figure 2F; 103% of sham, *p* = 0.95). In contrast, in aged animals, Aβ levels were greatly increased, at 3 days (143%, *p* = 0.0046), 3 weeks (180%, *p* = 0.0046) and 4 weeks (170%, *p* = 0.0046) after injection, compared to sham (Figure 2F). The antibody used to detect Aβ is not able to discriminate between human and mouse, however, due to this large difference in Aβ levels between the 3-month and 16 + month detected at the 4 week time-point, we hypothesize that the elevated Aβ levels observed in aged animals must reflect endogenously produced protein.

### AβO-injected Aged mice (16 + months) vs. Sham

#### Neurogenesis at 4 weeks after the AβO-lesion in 16 + month animals

**FIGURE 3:**
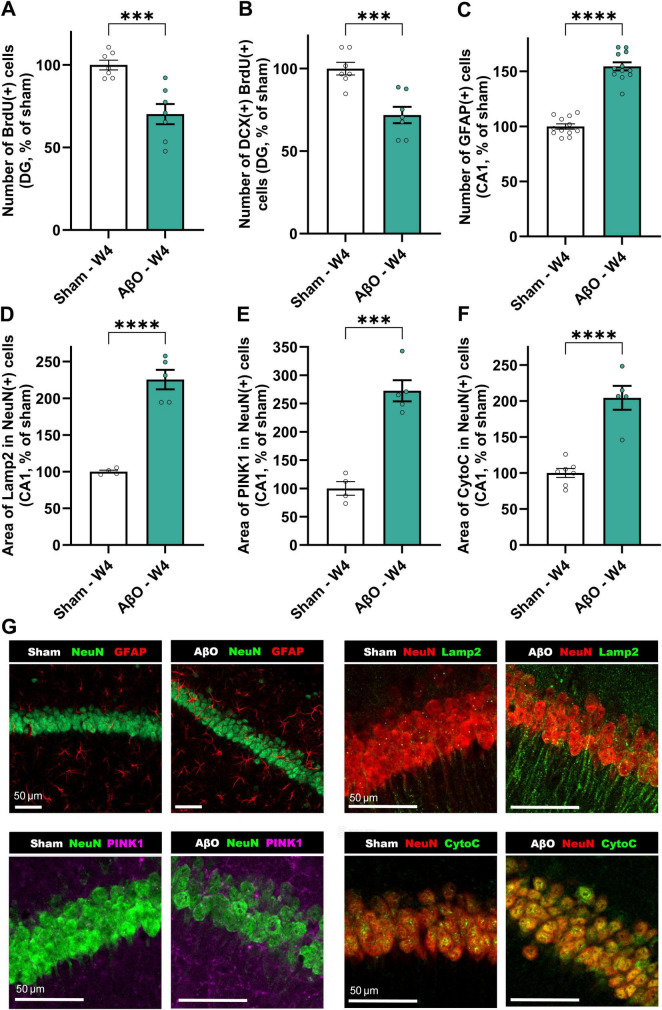
Histological and biochemical analysis of aged animals injected with Aβ compared to sham at 4 weeks post-injection. Neuroplasticity markers; **(A)** BrdU + cells in the DG [*n = 7*, normalized to sham where 100% = 14.45 ± 0.4 cells], **(B)** DCX/BrdU double-positive cells in the DG [*n = 7*, normalized to sham where 100% = 1.77 ± 0.1 cells]. **(C)** GFAP-positive astrocytes [*n = 12*, normalized to sham where 100% = 49.29 ± 2.22 cells]. Cellular stress markers; **(D)** area of Lamp2 per neuron [*n* = 4−5, normalized to sham where 100% = 245.69 ± 21.68 μm^2^], **(E)** area of PINK1 per neuron [*n* = 4−5, normalized to sham where 100% = 177.88 ± 21.66 μm^2^], and **(F)** area of CytoC per neuron [*n* = 5−7, normalized to sham where 100% = 152.21 ± 9.05 μm^2^]. Mean ± SEM, unpaired *t*-test. *p < 0.05, **p < 0.01, ***p < 0.001 and ****p < 0.0001. Representative images **(G)**; (i) NeuN (green) and GFAP (red), (ii) NeuN (red) and Lamp2 (green), (iii) NeuN (green) and PINK1 (pink), and (iv) NeuN (red) and CytoC (green) staining. Scale bars represent 50 μm.

To assess the impact of AβO injection in the CA1 region on neurogenesis, we evaluated the number of BrdU-positive cells, a marker of proliferating cells, and the proportion of these cells that expressed doublecortin (DCX), a marker of immature neurons, in the dentate gyrus (DG) at 4 weeks post-injection. In aged mice (16 + months), AβO injection significantly reduced the number of BrdU-positive cells in the dentate gyrus of the hippocampus, compared to sham-treated controls (Figure 3A; 70% of sham, *p* = 0.0009), indicating impaired proliferation or survival of newly generated cells. Furthermore, of the BrdU-positive cells that were present, a lower percentage co-expressed DCX (Figure 3B; 72% of sham, *p* = 0.0008), suggesting a skew away from neuronal differentiation in the aged, AβO-injected brain.

#### Neuroinflammation, lysosomal burden and mitochondrial stress at 4 weeks after AβO-injection—immunohistochemical analysis

As described in Figure 2C, levels of microglial proliferation are substantially elevated at 4 weeks-post-injury. Additionally, this AD-like model demonstrates greatly increased astrogliosis (Figure 3C; 155% of sham, *p* < 0.0001), as measured by GFAP expression, a marker of astrocytes. The aged AβO-injected mice also show significantly elevated levels of mitophagy marker, PINK1 (Figure 3E; 273% of sham, *p* = 0.0002), and mitochondrial stress marker, CytoC (Figure 3F; 172% of sham, *p* < 0.0001), as well as the lysosomal marker Lamp2 (Figure 3D; 226% of sham, *p* < 0.0001), within neurons in the CA1, compared to sham. Taken together, these results can be interpretated as an increase in cellular stress and impaired autophagic clearance in neurons following AβO exposure.

#### Spreading of pathology from the site of AβO injection site in aged mice

**FIGURE 4:**
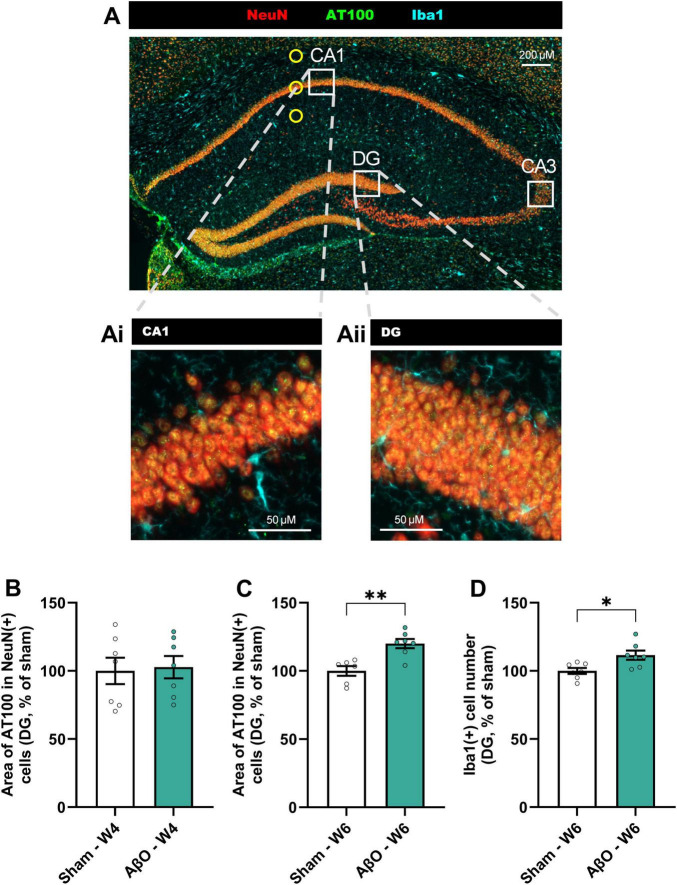
Spreading of pTau from CA1 to DG of hippocampus in Aβ-injected aged mice. **(A)** Representative image Axioscan (cropped), with white squares illustrating the analysis zones in the CA1 and DG regions (CA3 also illustrated to refer to Figure 5). Representative tiles in CA1 (Ai) and DG (Aii) selected for automated IHC analysis (size = 320 μm^2^); NeuN (red), pTau (AT100, green), and Iba1 (cyan). Scale bars indicate 50 μm. Quantitative analysis in the DG of **(B)** the area of AT100 in NeuN + cells at week 4 **(B)** [*n = 7*, normalized to sham where 100% = 41.6 ± 4.0 μm^2^] and week 6 **(C)** [*n* = 6−7, normalized to sham where 100% = 44.2 ± 1.6 μm^2^], and Iba1 staining at week 6 **(D)** [*n = 7*, normalized to sham where 100% = 135.0 ± 3.0 cells]. Mean ± SEM, unpaired *t*-test. *p < 0.05, **p < 0.01, ***p < 0.001 and ****p < 0.0001.

**FIGURE 5:**
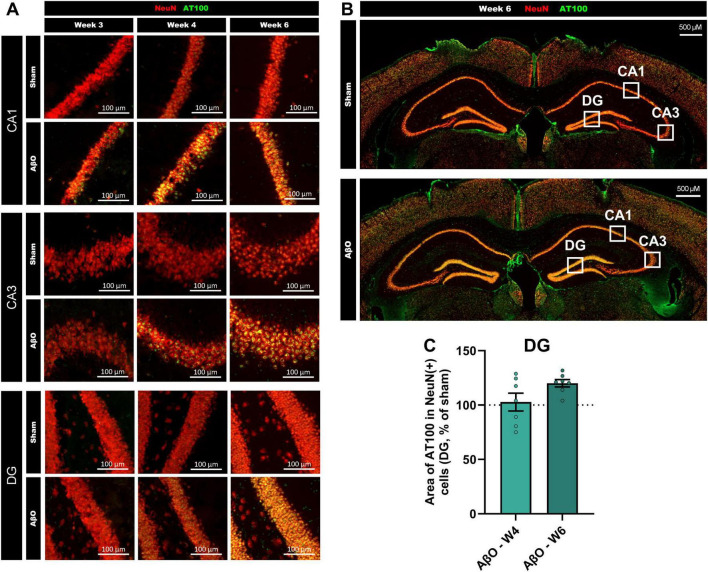
Progression of pTau spreading across the hippocampus over weeks post-Aβ -injection in aged mice. **(A)** Representative Axioscan images comparing pTau staining in the CA1 (upper panel), CA3 (center panel) and DG (lower panel), at week 3 (left), week 4 (central) and week 6 (right). pTau staining (AT100, green) and NeuN staining (red) are shown for both Aβ-injected mice and sham-injected controls. Scale bars indicate 100 μm. **(B)** Representative Axioscan entire images, for sham (above) and Aβ-treated (below) brains, example analysis zones marked with white squares. Scale bars indicate 500 μm. **(C)** Comparison of pTau levels in the DG at week 4 and 6 (*n* = 6-7).

Though Aβ was only injected into a specific region of the CA1, pathological changes were evident in other areas of the hippocampus (see Figure 4A for schematic and Figures 4, 5 for representative images). Images taken at week 3, 4, and 6 post-injection suggest that the pTau staining intensity is at first only evident in the CA1, then follows the route of pyramidal neuronal projections, becoming more intense next in the CA3, and then in the DG (Figure 5). Quantitative analysis reveals that, whilst the increased pTau accumulation in the CA1 was evident from 2 weeks post-injection (Figure 2D), further from the injection site, in the DG, an increase in pTau was only observed at 6 weeks post-injection (Figure 4C; 120% of sham, *p* = 0.02), whereas at 4 week there was no difference compared with sham (Figure 4B; 103%, *p* = 0.8). These time points are compared in Figure 5C. In addition, Iba1 was increased in the DG at 6 weeks post-injection (Figure 4D; 112%, *p* = 0.01).

## Discussion

This *in vivo* model recapitulates various features of AD, with neuronal mitochondrial stress (PINK1 and CytoC), lysosomal burden (Lamp2) and hyperphosphorylated tau accumulation, leading to a substantial loss of synapses and neuronal death. This is closely associated with astrogliosis (GFAP) and microglial activation (Iba1). Mitochondrial stress, mitophagy defects, loss of plasticity and synaptogenesis, associated with basal elevation of inflammation, are all features common to aging and AD. With these commonalities, an initial insult—whether an injury, toxin, or misfolded protein such as amyloid-beta (Aβ)—acts as the “seed,” capable of initiating a pathology that can flourish into progressive neurodegeneration.

We proved here that with age, deficits in short term memory were associated with a loss of hippocampal synapses and inflammation; because this soil is favorable, a stress can trigger a cascade of events leading to a pathological situation. Indeed, in a young brain, in addition to maintained homeostasis (such as low or non-existent oxidative stress and contained basal inflammation as we have shown), compensatory mechanisms exist and are highly effective. Here, we showed that neurogenesis was highly evident in hippocampi of young animals, compared to aged animals where it was drastically lower. Indeed, this plasticity can compensate for stress or any underlying lesion.

Injury to the young brain has been reported to induce signs of repair and formation of new connections in neurons proximal and distal to the damaged site (Lledo and Gheusi, 2006; Schoch et al., 2012). These compensatory mechanisms include circuit reorganization, neurogenesis, axonal growth, and dendritic plasticity, i.e., synaptogenesis (Nudo, 2007; Conner et al., 2005). Our findings show the dynamic nature of a young brain make it able to adapt to deleterious or stress events. Here after the amyloid infusion, some cognitive dysfunction associated with neuronal degeneration (at the site of injection) and inflammation were observed soon after the stress. However, all these defects disappeared with time and finally, a few weeks after the lesion, all observed impacts of stress were resolved via these compensatory mechanisms.

Interestingly, these compensatory brain mechanisms are also put in place in AD patients, in defense of the protein aggregation and amyloid spread (Bobkova and Vorobyov, 2015), but they become inadequate and eventually fail. In addition, as shown in this study, in old brains (16 m +) neurogenesis and synaptogenesis mechanisms were highly decreased (Figure 1), and even worse after amyloid stress (Figures 2, 3), rendering the compensatory mechanisms impossible.

The most accepted model indicates that Aβ pathophysiology is the upstream pathophysiological event in AD, triggering downstream molecular pathways, including tau misfolding, tau-mediated toxicity, accumulation in tangles, and tau spreading. This Tau pathology leads to cortical neurodegeneration and cognitive decline (He et al., 2018). Blocking Aβ production in cultures (using β- or γ-secretase inhibitors) prevented formation of tau pathology (Israel et al., 2012; Tokutake et al., 2012). In addition, application of AβO on primary culture induced hyperphosphorylation of tau and aggregation as well as activation of GSK3b (Callizot et al., 2013). By this rationale, the infusion of AβO is relevant to study the pathophysiology of AD.

Misfolded tau could spread neuron to neuron by a “prion-like” mechanism (Jucker and Walker, 2018). Similar phenomena have been implicated in other diseases (e.g., Parkinson’s disease, Henriques et al., 2022). Importantly, the spread of tau pathology into association cortices is nearly always associated with the presence of widespread amyloid plaques (Pontecorvo et al., 2017). Similarly, in the present study, Aβ infused in CA1 region induced a significant increase of pTau in the neurons surrounding the lesion, progressively expanding to neighboring cells, leading to tau propagation over the CA3 and DG.

This progression into other regions of the hippocampus overlapped with microglial activation (Iba1). It has been shown that an overexpression of tau can lead to microglial activation (even preceding tangle formation (Yoshiyama et al., 2007). Microglial cells may play a key role in the tau spreading either actively by transportation or passively by a deficit in phagocytosis, impairing clearance of pTau (Hopp et al., 2018). We cannot exclude that other mechanisms are also involved in the spreading, such as via extracellular vesicles in interneurons (as suggested by Ruan et al., 2021). The preliminary evidence here suggests that the spreading is mediated through neuronal projections, from the primary lesion area in the CA1, to the CA3 and then later the DG. In this study we focused on the hippocampus due to its importance in AD and memory, but we have also observed, in later stages after the lesion, some AT100 signal in regions outside of the hippocampus (as suggested in Figure 5B, in cortical and sub-cortical areas). This merits further investigation.

Here, we showed that several weeks after amyloid infusion, Aβ remained at in increased level in the hippocampus of aged brains (unlike in young brains where Aβ returns to sham levels 4 weeks after lesion). This increased Aβ supports the co-pathogenic interaction with tau in the disease progression (Pooler et al., 2015; Busche and Hyman, 2020; Gallego-Rudolf et al., 2024). A strong interrelationship exists between these 2 effectors (“trigger and bullet,” Bloom, 2014), however, the mechanisms behind Aβ and tau interplay in AD remain elusive (Roda et al., 2022).

All experiments on aged mice presented here have been performed on a colony of male mice, due to the technical reason that the availability of female mice of this age is greatly limited. However, sex-differences would be important to characterize, and these experiments are ongoing. Indeed, differences in the progression and severity of the disease have been observed in transgenic AD models (Melnikova et al., 2016; Zhong et al., 2024).

Our findings reinforce the notion that neurodegenerative processes do not act in isolation but are profoundly influenced by the underlying health of the brain. In our earlier studies on Parkinson’s disease (Henriques et al., 2022), we demonstrated that alpha-synuclein (αSyn) injected in *Substantia nigra pars compacta* (SNpc), combined with low-level impairments in lysosomal clearance, provides the spark that triggers a growing neurodegenerative fire in the aging brain. Here, we propose that Aβ acts in a similar way. The spread of inflammation in our study parallels observations in αSyn pathology in Parkinson’s disease models, where aged SNpc neurons also create fertile ground for protein aggregation and degeneration.

Beyond Aβ, tau, or αSyn, it is the environment within which these proteins accumulate that determines the progression and severity of neurodegeneration. This convergence of pathological mechanisms across neurodegenerative diseases points to a common factor: the aged, stressed brain, opening new possibilities for therapeutic interventions that go beyond targeting individual disease mechanisms to promote overall brain health.

## Data Availability

The raw data supporting the conclusions of this article will be made available by the authors, without undue reservation.

## Footnotes

1
https://www.proteinsimple.com/wes.html

## References

[B1] Antignano I. Liu Y. Offermann N. Capasso M. (2023). Aging microglia. *Cell. Mol. Life Sci.* 80:126. 10.1007/s00018-023-04775-y 37081238 PMC10119228

[B2] Bloom G. S. (2014). Amyloid-β and tau: The trigger and bullet in Alzheimer disease pathogenesis. *JAMA Neurol.* 71 505–508. 10.1001/jamaneurol.2013.5847 24493463 PMC12908160

[B3] Bobkova N. Vorobyov V. (2015). The brain compensatory mechanisms and Alzheimer’s disease progression: A new protective strategy. *Neural Regeneration Res.* 10 696–697. 10.4103/1673-5374.156954 26109935 PMC4468752

[B4] Bowling A. C. Mutisya E. M. Walker L. C. Price D. L. Cork L. C. Beal M. F. (1993). Age-dependent impairment of mitochondrial function in primate brain. *J. Neurochem*. 60, 1964–1967. 10.1111/j.1471-4159.1993.tb13430.x 8473911

[B5] Busche M. A. Hyman B. T. (2020). Synergy between amyloid-β and tau in Alzheimer’s disease. *Nat. Neurosci.* 23 1183–1193. 10.1038/s41593-020-0687-6 32778792 PMC11831977

[B6] Cai W. Li L. Sang S. Pan X. Zhong C. (2023). Physiological roles of β-amyloid in regulating synaptic function: Implications for AD pathophysiology. *Neurosci. Bull.* 39 1289–1308. 10.1007/s12264-022-00985-9 36443453 PMC10387033

[B7] Callizot N. Combes M. Steinschneider R. Poindron P. (2013). Operational dissection of β-amyloid cytopathic effects on cultured neurons. *J. Neurosci. Res.* 91 706–716. 10.1002/jnr.23193 23404368

[B8] Callizot N. Estrella C. Burlet S. Henriques A. Brantis C. Barrier M. (2021). AZP2006, a new promising treatment for Alzheimer’s and related diseases. *Sci. Rep.* 11:16806. 10.1038/s41598-021-94708-1 34413330 PMC8376949

[B9] Carmona-Gutierrez D. Hughes A. L. Madeo F. Ruckenstuhl C. (2016). The crucial impact of lysosomes in aging and longevity. *Ageing Res. Rev.* 32 2–12. 10.1016/j.arr.2016.04.009 27125853 PMC5081277

[B10] Conner J. M. Chiba A. A. Tuszynski M. H. (2005). The basal forebrain cholinergic system is essential for cortical plasticity and functional recovery following brain injury. *Neuron* 46 173–179. 10.1016/j.neuron.2005.03.003 15848797

[B11] Dhapola R. Beura S. K. Sharma P. Singh S. K. HariKrishnaReddy D. (2024). Oxidative stress in Alzheimer’s disease: Current knowledge of signaling pathways and therapeutics. *Mol. Biol. Rep.* 51:48. 10.1007/s11033-023-09021-z 38165499

[B12] Forner S. Kawauchi S. Balderrama-Gutierrez G. Kramár E. A. Matheos D. P. Phan J. (2021). Systematic phenotyping and characterization of the 5xFAD mouse model of Alzheimer’s disease. *Sci. Data* 8:270. 10.1038/s41597-021-01054-y 34654824 PMC8519958

[B13] Gallego-Rudolf J. Wiesman A. I. Pichet Binette A. Villeneuve S. Baillet S. Prevent-Ad Research (2024). Synergistic association of Aβ and tau pathology with cortical neurophysiology and cognitive decline in asymptomatic older adults. *Nat. Neurosci.* 27 2130–2137. 10.1038/s41593-024-01763-8 39294489 PMC11537964

[B14] Hampel H. Hardy J. Blennow K. Chen C. Perry G. Kim S. H. (2021). The Amyloid-β pathway in Alzheimer’s disease. *Mol. Psychiatry* 26 5481–5503. 10.1038/s41380-021-01249-0 34456336 PMC8758495

[B15] He Z. Guo J. L. McBride J. D. Narasimhan S. Kim H. Changolkar L. (2018). Amyloid-β plaques enhance Alzheimer’s brain tau-seeded pathologies by facilitating neuritic plaque tau aggregation. *Nat. Med.* 24 29–38. 10.1038/nm.4443 29200205 PMC5760353

[B16] Henriques A. Rouvière L. Giorla E. Farrugia C. El Waly B. Poindron P. (2022). Alpha-synuclein: The spark that flames dopaminergic neurons, in vitro and in vivo evidence. *Int. J. Mol. Sci.* 23:9864. 10.3390/ijms23179864 36077253 PMC9456396

[B17] Hong H. Kim B. S. Im H. I. (2016). Pathophysiological role of neuroinflammation in neurodegenerative diseases and psychiatric disorders. *Int. Neurourol. J.* 20 S2–S7. 10.5213/inj.1632604.302 27230456 PMC4895907

[B18] Hopp S. C. Lin Y. Oakley D. Roe A. D. DeVos S. L. Hanlon D. (2018). The role of microglia in processing and spreading of bioactive tau seeds in Alzheimer’s disease. *J. Neuroinflamm.* 15:269. 10.1186/s12974-018-1309-z 30227881 PMC6145371

[B19] Israel M. A. Yuan S. H. Bardy C. Reyna S. M. Mu Y. Herrera C. (2012). Probing sporadic and familial Alzheimer’s disease using induced pluripotent stem cells. *Nature* 482 216–220. 10.1038/nature10821 22278060 PMC3338985

[B20] Javanmehr N. Saleki K. Alijanizadeh P. Rezaei N. (2022). Microglia dynamics in aging-related neurobehavioral and neuroinflammatory diseases. *J. Neuroinflamm.* 19:273. 10.1186/s12974-022-02637-1 36397116 PMC9669544

[B21] Jawhar S. Trawicka A. Jenneckens C. Bayer T. A. Wirths O. (2012). Motor deficits, neuron loss, and reduced anxiety coinciding with axonal degeneration and intraneuronal Aβ aggregation in the 5XFAD mouse model of Alzheimer’s disease. *Neurobiol. Aging* 33:196.e29–40. 10.1016/j.neurobiolaging.2010.05.027 20619937

[B22] Jucker M. Walker L. C. (2018). Propagation and spread of pathogenic protein assemblies in neurodegenerative diseases. *Nat. Neurosci.* 21 1341–1349. 10.1038/s41593-018-0238-6 30258241 PMC6375686

[B23] Kawas C. Gray S. Brookmeyer R. Fozard J. Zonderman A. (2000). Age-specific incidence rates of Alzheimer’s disease: The baltimore longitudinal study of aging. *Neurology* 54 2072–2077. 10.1212/wnl.54.11.2072 10851365

[B24] Lambert M. P. Barlow A. K. Chromy B. A. Edwards C. Freed R. Liosatos M. (1998). Diffusible, nonfibrillar ligands derived from Abeta1-42 are potent central nervous system neurotoxins. *Proc. Natl. Acad. Sci. U S A.* 95 6448–6453. 10.1073/pnas.95.11.6448 9600986 PMC27787

[B25] Lledo P. M. Gheusi G. (2006). Neurogenèse adulte: Aspects fondamentaux et potentiels thérapeutiques [Adult neurogenesis: from basic research to clinical applications]. *Bull. Acad. Natl. Med.* 190, 385–400; discussion 400-2. 17001868

[B26] Long J. Gao F. Tong L. Cotman C. W. Ames B. N. Liu J. (2009). Mitochondrial decay in the brains of old rats: Ameliorating effect of alpha-lipoic acid and acetyl-L-carnitine. *Neurochem. Res.* 34 755–763. 10.1007/s11064-008-9850-2 18846423 PMC2790461

[B27] Mangrulkar S. V. Wankhede N. L. Kale M. B. Upaganlawar A. B. Taksande B. G. Umekar M. J. (2023). Mitochondrial dysfunction as a signaling target for therapeutic intervention in major neurodegenerative disease. *Neurotox. Res*. 41, 708–729. 10.1007/s12640-023-00647-2 37162686

[B28] Melnikova T. Park D. Becker L. Lee D. Cho E. Sayyida N. (2016). Sex-related dimorphism in dentate gyrus atrophy and behavioral phenotypes in an inducible tTa:APPsi transgenic model of Alzheimer’s disease. *Neurobiol. Dis.* 96 171–185. 10.1016/j.nbd.2016.08.009 27569580 PMC6457346

[B29] Nixon R. A. Yang D. S. Lee J. H. (2008). Neurodegenerative lysosomal disorders: A continuum from development to late age. *Autophagy* 4 590–599. 10.4161/auto.6259 18497567

[B30] Nizami S. Hall-Roberts H. Warrier S. Cowley S. A. Di Daniel E. (2019). Microglial inflammation and phagocytosis in Alzheimer’s disease: Potential therapeutic targets. *Br. J. Pharmacol.* 176, 3515–3532. 10.1111/bph.14618 30740661 PMC6715590

[B31] Nudo R. J. (2007). Postinfarct cortical plasticity and behavioral recovery. *Stroke* 38 840–845. 10.1161/01.STR.0000247943.12887.d2 17261749

[B32] Orr M. E. Oddo S. (2013). Autophagic/lysosomal dysfunction in Alzheimer’s disease. *Alzheimers Res. Therapy* 5:53. 10.1186/alzrt217 24171818 PMC3979020

[B33] Passarella S. Kethiswaran S. Brandes K. Tsai I. C. Cebulski K. Kröger A. (2024). Alteration of cGAS-STING signaling pathway components in the mouse cortex and hippocampus during healthy brain aging. *Front. Aging Neurosci.* 16:1429005. 10.3389/fnagi.2024.1429005 39149145 PMC11324507

[B34] Peters A. Sethares C. Luebke J. I. (2008). Synapses are lost during aging in the primate prefrontal cortex. *Neuroscience* 152 970–981. 10.1016/j.neuroscience.2007.07.014 18329176 PMC2441531

[B35] Pontecorvo M. J. Devous M. D. Navitsky M. Lu M. Salloway S. Schaerf F. W. (2017). Relationships between flortaucipir PET tau binding and amyloid burden, clinical diagnosis, age and cognition. *Brain* 140 748–763. 10.1093/brain/aww334 28077397 PMC5382945

[B36] Pooler A. M. Polydoro M. Maury E. A. Nicholls S. B. Reddy S. M. Wegmann S. (2015). Amyloid accelerates tau propagation and toxicity in a model of early Alzheimer’s disease. *Acta Neuropathol. Commun.* 3:14. 10.1186/s40478-015-0199-x 25853174 PMC4371800

[B37] Roda A. R. Serra-Mir G. Montoliu-Gaya L. Tiessler L. Villegas S. (2022). Amyloid-beta peptide and tau protein crosstalk in Alzheimer’s disease. *Neural Regeneration Res.* 17 1666–1674. 10.4103/1673-5374.332127 35017413 PMC8820696

[B38] Ruan Z. Pathak D. Venkatesan Kalavai S. Yoshii-Kitahara A. Muraoka S. Bhatt N. (2021). Alzheimer’s disease brain-derived extracellular vesicles spread tau pathology in interneurons. *Brain* 144 288–309. 10.1093/brain/awaa376 33246331 PMC7880668

[B39] Schoch K. M. Madathil S. K. Saatman K. E. (2012). Genetic manipulation of cell death and neuroplasticity pathways in traumatic brain injury. *Neurotherapeutics* 9 323–337. 10.1007/s13311-012-0107-z 22362424 PMC3337028

[B40] Shankar G. M. Walsh D. M. (2009). Alzheimer’s disease: Synaptic dysfunction and Abeta. *Mol. Neurodegener.* 4:48. 10.1186/1750-1326-4-48 19930651 PMC2788538

[B41] Tokutake T. Kasuga K. Yajima R. Sekine Y. Tezuka T. Nishizawa M. (2012). Hyperphosphorylation of Tau induced by naturally secreted amyloid-β at nanomolar concentrations is modulated by insulin-dependent Akt-GSK3β signaling pathway. *J. Biol. Chem.* 287 35222–35233. 10.1074/jbc.M112.348300 22910909 PMC3471719

[B42] Vossel K. A. Xu J. C. Fomenko V. Miyamoto T. Suberbielle E. Knox J. A. (2015). Tau reduction prevents Aβ-induced axonal transport deficits by blocking activation of GSK3β. *J. Cell Biol.* 209 419–433. 10.1083/jcb.201407065 25963821 PMC4427789

[B43] Woodburn S. C. Bollinger J. L. Wohleb E. S. (2021). The semantics of microglia activation: Neuroinflammation, homeostasis, and stress. *J. Neuroinflammation* 18:258. 10.1186/s12974-021-02309-6 34742308 PMC8571840

[B44] Yoshiyama Y. Higuchi M. Zhang B. Huang S. M. Iwata N. Saido T. C. (2007). Synapse loss and microglial activation precede tangles in a P301S tauopathy mouse model. *Neuron* 53 337–351. 10.1016/j.neuron.2007.01.010 17270732

[B45] Zhong M. Z. Peng T. Duarte M. L. Wang M. Cai D. (2024). Updates on mouse models of Alzheimer’s disease. *Mol. Neurodegener.* 19:23. 10.1186/s13024-024-00712-0 38462606 PMC10926682

