# Supplementary material for: Amyloid-beta peptide toxicity in the aged brain is a one-way journey into Alzheimer’s disease
Source: Front Aging Neurosci. 2025 Apr 30;17:1569181. doi: 10.3389/fnagi.2025.1569181 (PMC12075133; doi:10.3389/fnagi.2025.1569181)
Supplement: Supplementary file 1 [file Table_1.docx]

**Supp. Table 1; List of primary antibodies used for immunohistochemistry**

| **Antibodies** | **Source** | **Catalog Number** |
| --- | --- | --- |
| Chicken polyclonal NeuN | Abcam | Ab134014 |
| Rabbit polyclonal PSD95 | Ozyme | 2507S |
| Rabbit monoclonal GAPDH | Ozyme | 2118S |
| Mouse monoclonal 6E10 | BioLegend | 803001 |
| Mouse monoclonal AT100 | Fisher Scientific | 11818711 |
| Rabbit polyclonal BrdU | Sigma Aldrich | HMBJ2225 |
| Rabbit polyclonal DCX | Ozyme | 4604S |
| Rabbit polyclonal GFAP | Sigma Aldrich | G9269 |
| Chicken polyclonal GFAP | Abcam | Ab254083 |
| Rabbit polyclonal Lamp2 | Fisher Scientific | 10332473 |
| Mouse monoclonal PINK1 | Abcam | Ab186303 |


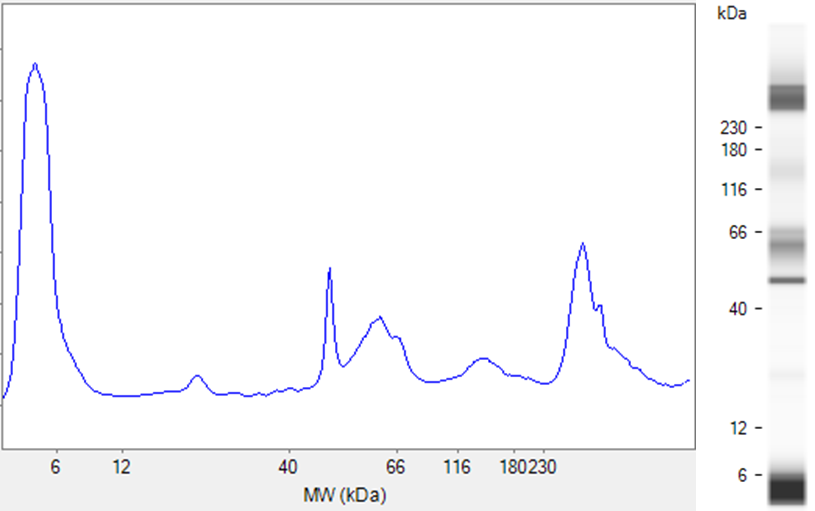


**Supp. Fig. 1 ; Quantification of entities in Aβ-preparation;** An example trace of the automated protein analysis (by WES) as performed on each batch of Aβ solution to be injected, to ensure identical proportions of monomeric, oligomeric and protofibril/fibril fractions.


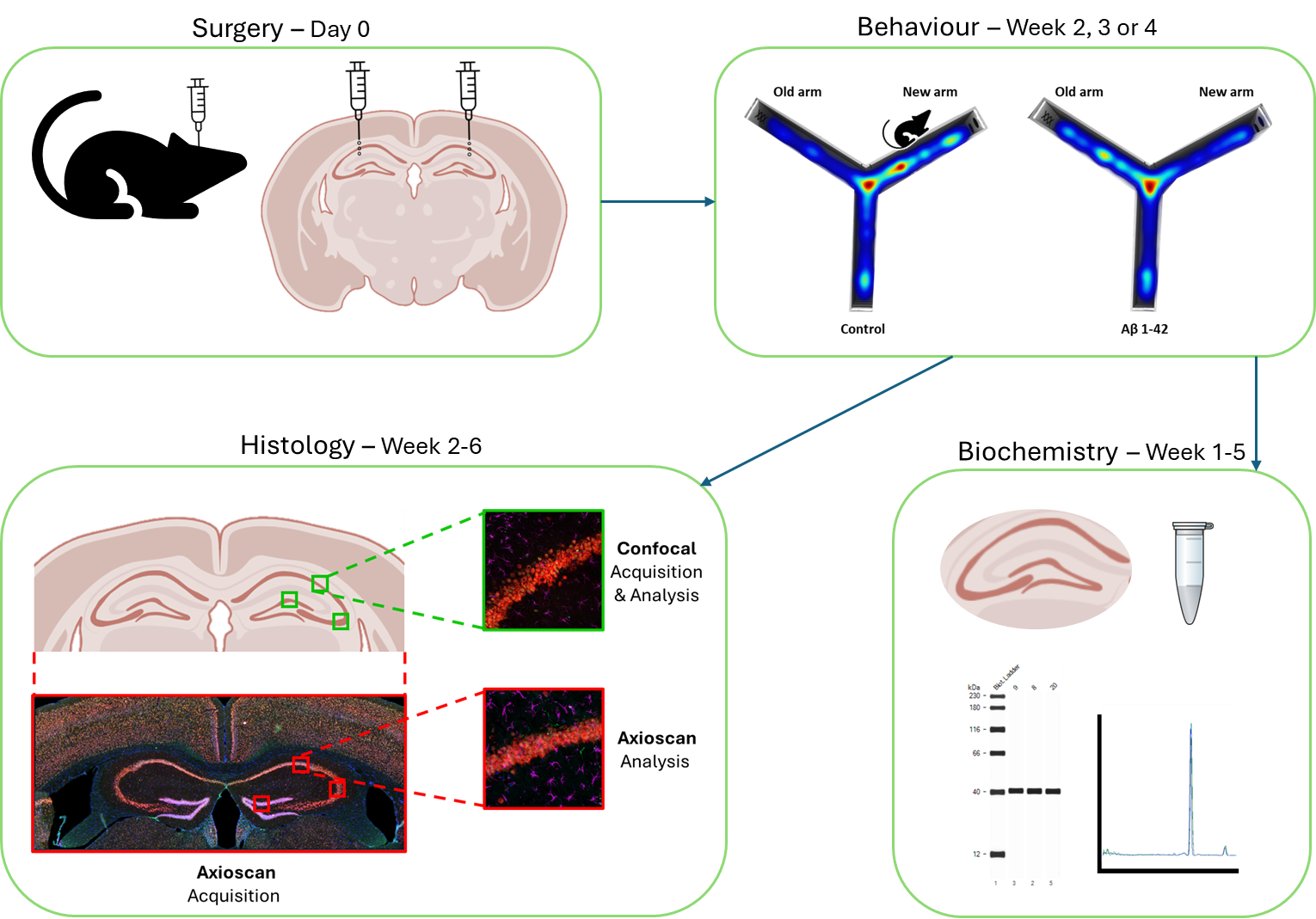


**Supp. Fig. 2 ; Schematic representation of experiments, from surgery to behavior, then histology or biochemistry.** A; Stereotaxic bi-lateral injection site in at 3 depths (black circles) in the hippocampal CA1. B; Y-maze automated analysis. C; Histological analysis zones for confocal (green) and Axioscan (red). Confocal microscopy; Image acquisition and analysis performed directly in the CA1 or DG. Axioscan; Image acquisition encompasses both hippocampi, then analysis zones later selected in the CA1 (150 µm from injection site), CA3 or DG cell body layer. D; Protein levels in whole hippocampi assessed WES automated western blotting and analysis.


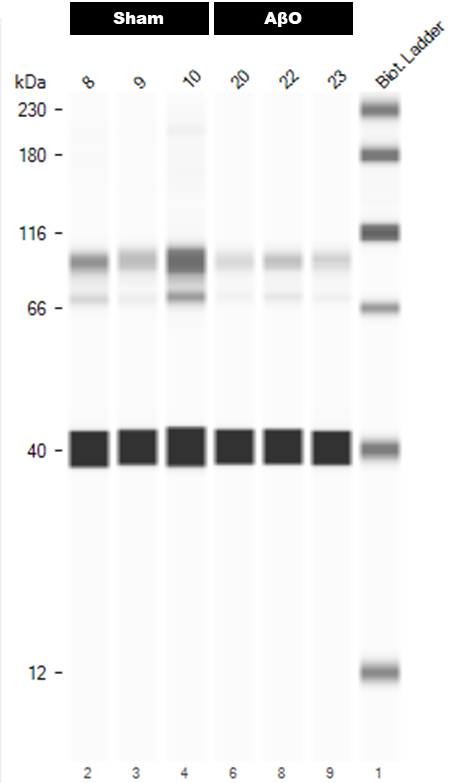


**Supp. Fig. 3; Quantification of PSD95 levels;** An example trace of the automated protein analysis (by WES) for sham versus Aβ-treated.


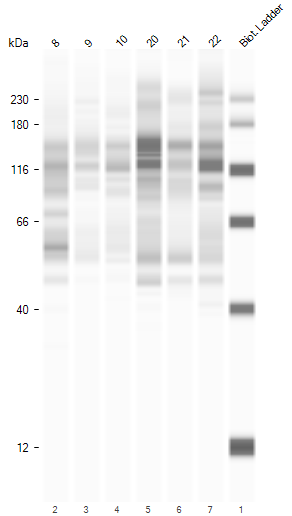


AβO

Sham

**Supp. Fig. 4; Quantification of Aβ (6E10) levels;** An example trace of the automated protein analysis (by WES) for sham versus Aβ-treated.
